# Revisiting the disabilities of the arm, shoulder and hand (DASH) and QuickDASH in rheumatoid arthritis

**DOI:** 10.1186/s12891-019-2414-6

**Published:** 2019-01-25

**Authors:** B. Prodinger, A. Hammond, A. Tennant, Y. Prior, S. Tyson

**Affiliations:** 1 Swiss Paraplegic Research, Nottwil, Switzerland; 2 Department of Health Sciences and Health Policy, University of Lucerne, Lucerne, Switzerland; 3 ICF Research Branch, a cooperation partner within the WHO Collaborating Centre for the Family of International Classifications in Germany (at DIMDI), Nottwil, Switzerland; 4 Faculty of Applied Health and Social Sciences, Technical University of Applied Sciences Rosenheim, Rosenheim, Germany; 5 Centre for Health Sciences Research, School of Health and Society, University of Salford, Salford, UK; 6 Division of Nursing, Midwifery & Social Work, University of Manchester, Manchester, UK; 7 Mid Cheshire NHS Trust Foundation Hospitals, Leighton Hospital, Crewe, UK

## Abstract

**Background:**

Limitations in upper limb functioning are common in Musculoskeletal disorders and the Disabilities of the Arm, Shoulder and Hand scale (DASH) has gained widespread use in this context. However, various concerns have been raised about its construct validity and so this study seeks to examine this and other psychometric aspects of both the DASH and QuickDASH from a modern test theory perspective.

**Methods:**

Participants in the study were eligible if they had a confirmed diagnosis of Rheumatoid Arthritis (RA). They were mailed a questionnaire booklet which included the DASH. Construct validity was examined by fit to the Rasch measurement model. The degree of precision of both the DASH and QuickDASH were considered through their Standard Error of Measurement (SEM).

**Results:**

Three hundred and thirty-seven subjects with confirmed RA took part, with a mean age of 62.0 years (SD12.1); 73.6% (*n* = 252) were female. The median standardized score on the DASH was 33 (IQR 17.5–55.0). Significant misfit of the DASH and QuickDASH was observed but, after accommodating local dependency among items in a two-testlet solution, satisfactory fit was obtained, supporting the unidimensionality of the total sets and the sufficiency of the raw (ordinal or standardized) scores.

**Conclusion:**

Having accommodated local response dependency in the DASH and QuickDASH item sets, their total scores are shown to be valid, given they satisfy the Rasch model assumptions. The Rasch transformation should be used whenever all items are used to calculate a change score, or to apply parametric statistics within an RA population.

**Significance and innovations:**

- Most previous modern psychometric analyses of both the DASH and QuickDASH have failed to fully address the effect of a breach of the local independence assumption upon construct validity.
- Accommodating this problem by creating ‘super items’ or testlets, removes this effect and shows that both versions of the scale are valid and unidimensional, as applied with a bi-factor equivalent solution to an RA population.
- The Standard Error of Measurement of a scale can be biased by failing to take into account the local dependency in the data which inflates reliability and thus making the SEM appear better (i.e. smaller) than the true value without bias.

**Electronic supplementary material:**

The online version of this article (10.1186/s12891-019-2414-6) contains supplementary material, which is available to authorized users.

## Background

Limitations in upper limb functioning, and associated impact upon daily activities are common in musculoskeletal disorders, as well as many other long-term conditions. One recent large-scale survey in China reported prevalence of rheumatic pain in the neck, shoulder and elbow as 5.6, 3.1 and 1.4% respectively [1]. Another study in the USA, with a sample whose mean age was 68 years, found symptoms associated with the neck and shoulder to have a prevalence of 8 and 13% respectively [2]. An earlier large-scale study in those aged 16 years and above in the UK found the prevalence of pain, swelling or stiffness of shoulder, elbow and hand at 6.9, 3.1 and 6.6% respectively [3]. Thus, upper limb problems are common in the population, and particularly so among those with musculoskeletal disorders and in older people.

It is not surprising therefore that several Patient Reported Outcome Measures (PROMs) have been developed with the intention of assessing the extent of impairment of function, or of limitations in activities associated with the upper limb function in both children and adults [4, 5] . One such PROM, the Disabilities of the Arm, Shoulder and Hand scale (DASH) has gained widespread use across many chronic conditions [6, 7]. However, at the same time, concerns have been raised from a classical test theory perspective about the viability of the summated score of its 30 items, suggesting that the scale comprises more than one domain [8–10]. Similar concerns have been expressed from a modern test theory perspective when data from the scale have been fit to the Rasch model, indicating lack of dimensionality, a breach of the local independence assumption among its items, and lack of consistent scaling properties of its items [11–13]. Meanwhile, a shortened 11-item version, the QuickDASH, has emerged which has also been criticized from both test perspectives [14–16].

This study seeks to examine the internal construct validity and other psychometric aspects of both the DASH and QuickDASH, from a modern test theory perspective, in a population of those with Rheumatoid Arthritis.

## Methods

The National Research Ethics Service Committee North West - Greater Manchester North (12/NW/0841) and the University of Salford School of Health Sciences Ethics Panel provided ethical approval for this study. All participants provided written, informed consent.

### Participants

Two recruitment strategies were applied in parallel. Firstly, research nurses recruited in 17 Rheumatology out-patient clinics or identified participants from department databases. Secondly, participants willing to be contacted for future studies of a previously conducted outcome measure study from the same Rheumatology out-patient clinics originally were re-checked for eligibility prior to consent. The following eligibility criteria were applied: 1) confirmed diagnosis of rheumatoid arthritis (RA); 2) being able to read, write and understand English; and 3) had not (or were not about to) altered their disease-modifying medication regimen in the last three months.

### Procedures

A questionnaire booklet which included the information about the to be recruited study population: demographic and disease data: age, gender, marital, educational and employment status, disease duration and RA disease-modifying medication was mailed to participants.

### The DASH and QuickDASH scoring

The DASH consists of 30 items scored on a 1–5 scale. The scoring instructions indicate that summating all items to a total score is acceptable, given at least 27 items have been completed. An algorithm is available to construct an overall standardized score of 0–100, including coping with missing values. The 30-item scale can be said to assess upper limb functioning, comprising both aspects of pain, and activities of daily living. The QuickDASH is formed from a subset of 11 items, and is scored in a similar fashion. In the current study this was derived from participants’ completed DASH questionnaires.

### Construct validity

Construct validity was examined by fit to the Rasch measurement model [17]. Data were fit to the Rasch measurement model using the RUMM2030 software [18]. The Rasch model is widely used in health outcomes to determine the sufficiency of the raw score, unidimensionality, local independence, Differential Item Functioning (DIF), and the threshold ordering of polytomous items. Various published papers explain these aspects in some detail [19, 20]. In this study, fit to the model was determined through a non-significant Chi-Square (Benjamin-Hochberg adjusted *p* values with 25% false discovery rate) and DIF was evaluated for age and gender. Any breach of the local (response) independence assumption was tested through item residual correlations of ≥0.2 above the average residual correlation [21, 22]. Where this breach occurred, items were summated together into testlets to absorb the local dependency [23] (super items which simply add up the item set into one new item). When these testlets were used to assess the scale, additional indicators were available. Expressed as the value ‘A’ in the RUMM2030 program, this is the proportion of common non-error variance retained in the resulting latent estimate, and where a value of 0.85 and above is considered sufficient for supporting a strong unidimensional general factor, consistent with the Explained Common Variance (ECV) to be found in the bi-factor literature [24, 25].

### Precision

Finally, the degree of precision of both the DASH and QuickDASH were considered through their Standard Error of Measurement (SEM), and the Smallest Detectable Difference (SDD) expressed as a percentage of the operational range of the scale. The latter is concerned with that value which is needed to show a meaningful difference above the level of error in the scale, and is represented as 1.96* SEM.

## Results

### Participants

Three hundred and thirty-seven subjects with confirmed RA completed the DASH questionnaire, with a mean age of 62.0 years (SD12.1), 73.6% (*n* = 252) were female, 71.7% (*n* = 241) were married or living with a partner and 10.8% (*n* = 36) had children living at home. Over half (*n* = 169;50.8%) were retired, and a further 10.6% (*n* = 35) had retired early due to ill health.

### DASH scores

The median score on the DASH using the standardized scoring system (i.e. range 0–100) was 33 (IQR 17.5–55.0). Only eight participants (2.4%) had more than three missing responses. A significant difference was observed between DASH scores by gender, with females having a higher (worse) score than males (Mann-Whitney U Z score = − 2.609; *p* = 0.009).

### Rasch analysis

#### Dash-30

**Fig. 1:**
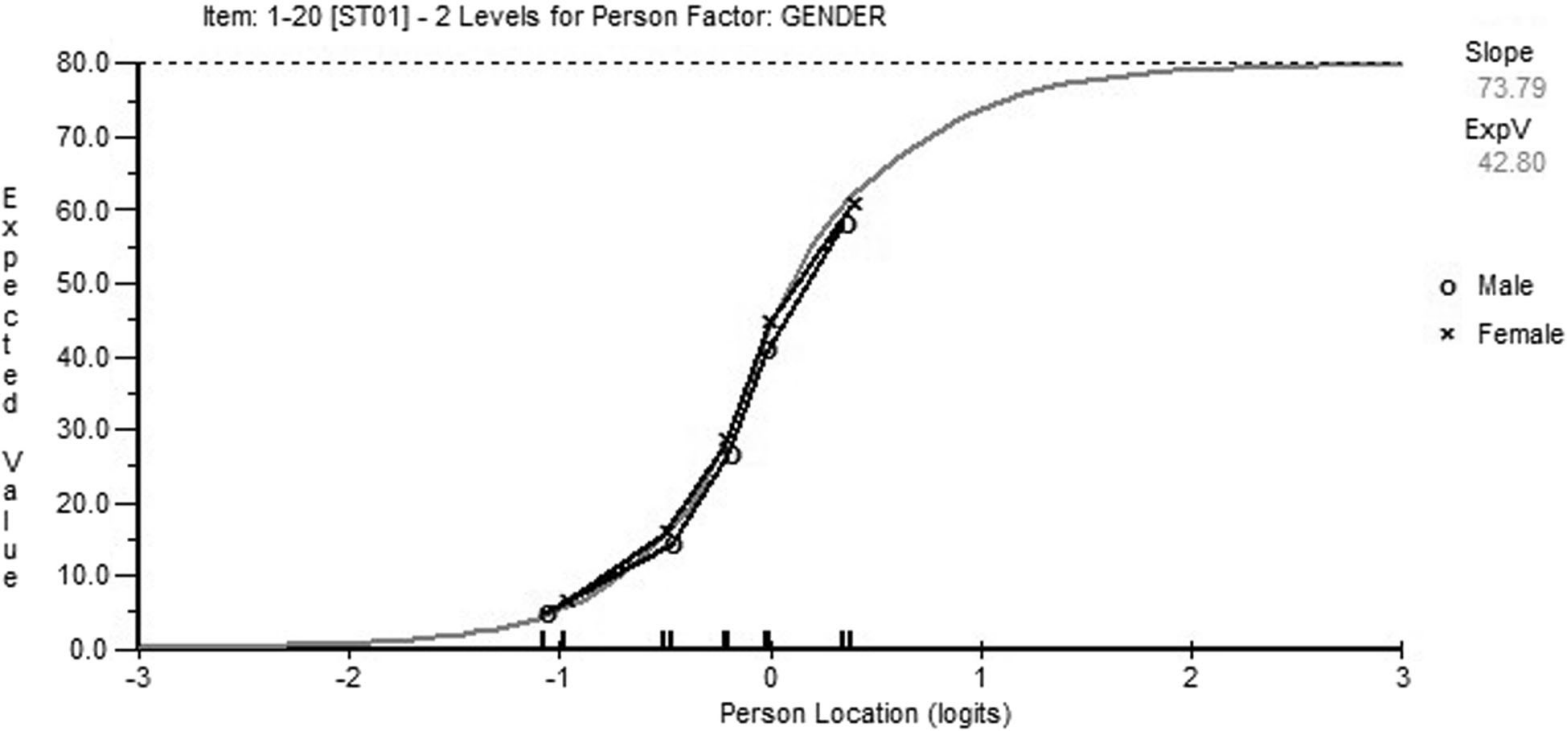
Differential item functioning by gender for PF testlet

The data from the 30 items were fit to the Rasch measurement model. The initial fit to support a unidimensional structure was poor (Chi-square = 518 (df 120) *p* = < 0.001; item fit residual SD 2.6; person fit residual SD 1.48; reliability (Person Separation Index (PSI) = 0.97). The local independence assumption was breached by 27 pairs of significant residual correlations (> 0.02), indicating a pattern consistent with two potential domains of activity limitations, incl. Mobility and self-care and impairments of functions such as pain. For example, the items “Recreational activities in which you take some force or impact through your arm, shoulder or hand (e.g., golf, hammering, tennis, etc.)” and “Recreational activities in which you move your arm freely (e.g., playing frisbee, badminton, etc.)” had a residual correlation of 0.472. The residual correlation matrix of the DASH-30 and QuickDASH can be found in the Additional file 1: Tables S1 and S2. Thus, taking the domains identified, two testlets (super items) were created to absorb the local dependency. In this instance fit showed a Chi-Square of 7.6 (df 8); *p* = 0.469; and reliability (Person Separation Index: PSI) of 0.85, which was satisfactory. However, DIF was present for gender across both testlets (but not for age). Both testlets displayed the same pattern, the DIF was present for the first class interval representing a group with least problems, and this magnitude did not exceed 0.11 logits. For higher levels of problems with, for example PF (testlet containing item 1-20), the magnitude of difference did not exceed 0.04 logits. The graphical interpretation of DIF on the PF testlet (items 1–20) is shown in Fig. 1. As such no action was taken for DIF.

The unidimensional latent estimate so derived accounted for 88% of the total non-error variance and was thus consistent with an ECV indicative of a strong general factor upon which all items load, supporting the unidimensionality of the total set, having accounted for the local dependency in the data. The t-test of the two independent estimates derived from the testlets showed just 2.2% of estimates were significantly different, supporting unidimensionality. This was further supported by the latent correlation of the two testlets being 0.97. No individual showed a positive fit residual > 0.124, (where 2.5 an above would indicate misfit) suggesting that the total score adequately represents the profile of responses in all cases.

#### QuickDASH

Fit of the 11 items of the QuickDASH to the Rasch model was poor (Chi-Square 221.4 (44); *p* < 0.001; Item fit residual SD 2.62; Person fit residual SD 1.12; reliability (PSI) = 0.92). Again, the pattern of residual correlations indicated two domains. Applying the two testlet solution, resulted in satisfactory fit (Chi-Square 10.9 (8); *p* = 0.205; reliability (PSI = 0.84; Cronbach’s α = 0.85). The t-test of the differences between the two testlet derived estimates showed that just 1.6% of estimates were different. The latent estimate indicated an ECV of 0.9. No person had a positive residual greater than 1.0, indicating that the raw score from all 11 items reflects the pattern of responses in all cases.

### Measurement precision

The SEM of the DASH (on a score range of 30–150) was 10.47, and that of the QuickDASH (on a score range of 11–55) was 3.95. The SDD of each was 29.0 and 10.9 respectively. This amounted to 24.2% of the operational range of the DASH, and 24.9% of the operational range of the QuickDASH. If the published figures on scale reliability were used, instead of those adjusted to avoid the bias of the local independence assumption, then the SEM of the DASH was 5.03, and that of the QuickDASH was 2.50. Consequently, the SDD would be 13.9 and 6.9, respectively, representing 11.6 and 15.7% of the scale widths.

### Exchange between DASH-30 and QuickDASH

Given fit to the Rasch model, any subset of items should give a valid estimate of the person’s upper-limb functioning. Consequently, two new testlets were created, one with the QuickDASH set of 11 items, the second with the remaining 19 items. Fit to the model was excellent (Chi-Square = 6.71 (df 8) *p* = 0.569; reliability (PSI = 0.96; Cronbach’s α = 0.89). the t-test of the differences between the two testlet derived estimates showed that just 5.3% (95%CI: 2.5–8.2) of estimates were different. The latent estimate indicated an ECV of 0.99, and the latent correlation between the two tests was 1.0. There was no DIF by age or gender. As such a transformation between the two versions of the scale was available. Table 1 gives the raw score (using the 0–4 range giving a total range of 0–120 (add 30 to create the 30–150 range), the standardized score for each scale (range 0–100), and the associated interval scale Rasch metric (range 0–100) through a simple linear rescaling of the ordinal raw score. The latter can be used for parametric analyses, assuming appropriate distributions.

**Table 1:** Raw score to interval scale Rasch Metric for DASH and QUICKDASH

DASH Raw	DASH	DASH	QuickDASH Raw	QuickDASH	DASH
(0–4)	Standard	Rasch	(0–4)	Standard	Rasch
0	0.0	0.0	0	0.0	0.0
1	0.8	5.5	1	2.3	6.4
2	1.7	9.1	2	4.5	11.5
3	2.5	11.5	3	6.8	15.6
4	3.3	13.3	4	9.1	19.4
5	4.2	14.9	5	11.4	23.0
6	5.0	16.3	6	13.6	26.4
7	5.8	17.6	7	15.9	29.6
8	6.7	18.9	8	18.2	32.7
9	7.5	20.1	9	20.5	35.5
10	8.3	21.3	10	22.7	38.2
11	9.2	22.5	11	25.0	40.7
12	10.0	23.6	12	27.3	43.0
13	10.8	24.7	13	29.5	45.2
14	11.7	25.8	14	31.8	47.3
15	12.5	26.8	15	34.1	49.2
16	13.3	27.9	16	36.4	51.0
17	14.2	28.9	17	38.6	52.8
18	15.0	29.9	18	40.9	54.4
19	15.8	30.8	19	43.2	55.9
20	16.7	31.8	20	45.5	57.4
21	17.5	32.7	21	47.7	58.8
22	18.3	33.6	22	50.0	60.1
23	19.2	34.5	23	52.3	61.4
24	20.0	35.4	24	54.5	62.7
25	20.8	36.2	25	56.8	63.9
26	21.7	37.0	26	59.1	65.1
27	22.5	37.8	27	61.4	66.4
28	23.3	38.6	28	63.6	67.6
29	24.2	39.4	29	65.9	68.9
30	25.0	40.2	30	68.2	70.1
31	25.8	40.9	31	70.5	71.4
32	26.7	41.6	32	72.7	72.8
33	27.5	42.3	33	75.0	74.3
34	28.3	43.0	34	77.3	75.8
35	29.2	43.7	35	79.5	77.3
36	30.0	44.3	36	81.8	79.0
37	30.8	45.0	37	84.1	80.7
38	31.7	45.6	38	86.4	82.6
39	32.5	46.2	39	88.6	84.5
40	33.3	46.8	40	90.9	86.6
41	34.2	47.4	41	93.2	88.9
42	35.0	48.0	42	95.5	91.5
43	35.8	48.6	43	97.7	95.1
44	36.7	49.1	44	100.0	100.0
45	37.5	49.7			
46	38.3	50.2			
47	39.2	50.7			
48	40.0	51.3			
49	40.8	51.8			
50	41.7	52.3			
51	42.5	52.8			
52	43.3	53.2			
53	44.2	53.7			
54	45.0	54.2			
55	45.8	54.7			
56	46.7	55.1			
57	47.5	55.6			
58	48.3	56.0			
59	49.2	56.5			
60	50.0	56.9			
61	50.8	57.3			
62	51.7	57.8			
63	52.5	58.2			
64	53.3	58.6			
65	54.2	59.1			
66	55.0	59.5			
67	55.8	59.9			
68	56.7	60.3			
69	57.5	60.7			
70	58.3	61.2			
71	59.2	61.6			
72	60.0	62.0			
73	60.8	62.4			
74	61.7	62.9			
75	62.5	63.3			
76	63.3	63.7			
77	64.2	64.1			
78	65.0	64.6			
79	65.8	65.0			
80	66.7	65.5			
81	67.5	65.9			
82	68.3	66.3			
83	69.2	66.8			
84	70.0	67.3			
85	70.8	67.7			
86	71.7	68.2			
87	72.5	68.7			
88	73.3	69.2			
89	74.2	69.6			
90	75.0	70.2			
91	75.8	70.7			
92	76.7	71.2			
93	77.5	71.7			
94	78.3	72.2			
95	79.2	72.8			
96	80.0	73.3			
97	80.8	73.9			
98	81.7	74.5			
99	82.5	75.1			
100	83.3	75.7			
101	84.2	76.3			
102	85.0	76.9			
103	85.8	77.6			
104	86.7	78.2			
105	87.5	78.9			
106	88.3	79.6			
107	89.2	80.3			
108	90.0	81.0			
109	90.8	81.8			
110	91.7	82.5			
111	92.5	83.3			
112	93.3	84.1			
113	94.2	85.0			
114	95.0	86.0			
115	95.8	87.1			
116	96.7	88.3			
117	97.5	89.8			
118	98.3	91.8			
119	99.2	95.0			
120	100.0	100.0			

An exchange between the two scales can be obtained by looking at either the raw score or the standardized score, and the associated Rasch metric. For example, consider a standardized score on the DASH of 40.0, and its associated Rasch metric of 51.3. The nearest equivalent Rasch value on the QuickDASH is 51.0, and this is associated with a QuickDASH standardized score of 36.4. Thus, a DASH standardized score of 40.0 is equal to a QuickDASH standardized score of 36.4.

## Discussion

Taking the basic 30-item (DASH) and 11-item (QuickDASH) scales, and applying modern test theory analyses showed that neither versions of the DASH satisfied model expectations. Multidimensionality, and a breach of the local (response) independent assumptions, were evident in both cases. Thus, the initial findings are consistent with the previously published psychometric evidence for the scales [8–16, 26]. Yet in both scales, clues exist as to why these findings may misinterpret the construct validity. This lies in the breach of the local independence assumption, whereby clusters of items can be found with varying degrees of residual correlation. In clinical scales this is quite common as, for example, in rehabilitation, health professionals may need to know whether or not a patient can dress their upper body, and their lower body, despite psychometric evidence showing a high residual correlation between the two activities. This type of effect of the breach of the local independence solution has been shown to both inflate classical reliability and to be corrosive for interpretation of the Rasch model [27]. Historically this has led to potential misinterpretation of the construct validity of well-known scales [28]. For example, it can make thresholds appeared disordered, and in the current analysis, of the 5 thresholds that were disordered, four were locally dependent with one or more other items. Local dependency can also drive multidimensionality, which was also the case here. Thus, the absence of applying suitable methods to accommodate this dependency may address some of the challenges identified in previously published findings about the scales.

Consequently, having applied the testlet approach to accommodating local response dependency in the DASH and QuickDASH item sets, within their frame of reference of rheumatoid arthritis, their total (ordinal) scores are shown to be valid given they both satisfy the Rasch model assumptions. It is possible that the frame of reference is crucial, as the DASH and QuickDASH are generic scales, and applied across a wide range of conditions. Thus previous findings in other conditions may reflect differences in how the scale works across different conditions [29]. However, a proper comparison cannot be made until the local dependency issue is firmly dealt with in those other conditions. Failure to take this into account may lead to erroneous conclusions about the construct validity of the scale, including the interpretation of multidimensionality driven by the local dependency. It may also, as shown above, lead to erroneous conclusions about the SEM and SDD of a scale. An inflated reliability will make a scale look much better than it really is, with a lower SEM and consequent SDD.

While the scale or level of detail is reduced in the testlet-based analysis, although this is consistent with how the scales are used in practice, examination at the item level in diagnostic mode is also informative. There is little doubt that the item ‘Tingling’ is a major threat to fit within Rasch model framework. In both the DASH and QuickDASH Rasch analyses, this item at the individual item level, showed a magnitude of misfit much greater than all other items, consistent with the findings of earlier studies [8]. Furthermore, unlike most other items which showed local dependency with one or more other items, ‘tingling’ showed no such dependency at all. Yet when absorbed into the more abstract ‘Impairment of Functions’ testlet, along with other items such as pain, the testlet itself shows no signs of misfit. So, perhaps the effect of the ‘tingling’ item is mitigated at this level, as indeed it must be at the whole test level. Thus, given the focus of use is always on the whole scale score (or subscales when present), the question arises as to the appropriate focus for analysis. The two testlet solution, as applied here, is almost at the whole scale level, similar to the classical test theory approach. Indeed, recent approaches in scale banking, whereby full scale scores for a particular construct are used as items, suggest a different approach to understanding of scale construct validity, albeit still from the Rasch Measurement Theory perspective [30]. Here the emphasis is upon the scale score as a whole, co-calibrated with other total scores from other scales measuring the same construct, which technically does not even need the items, as the total score becomes the item in such an approach. As such the focus of analysis becomes crucial. Where existing scales are being considered, their total (or subscale) score can be the initial focus, although if this fails, then the analysis at the item level sharpens as the reason for failure is explored. When new scales are being developed, then the focus should be reversed, and each individual item considered in the context of all others, and considered for inclusion in the final version of the scale.

While classical test theory approaches have failed to confirm the factor structure of the scales, it is possible that a bi-factor solution may have worked, as the ‘A’ value in the RUMM2030 software is equivalent to the ECV in the bi-factor literature, having been derived from the difference in the reliability estimates between the item- and testlet-based solutions [24]. As in the current study the ECV was below 1, it does suggest that some (although not much) of the variance was discarded in the latent estimate, which would populate the secondary factors in the classical bi-factor model. Nevertheless, both the DASH and QuickDASH had ECV values consistent with a dominant unidimensional first factor, and the associated t-test confirmed that the latent estimates so derived were unidimensional [31].

One caveat to the above analysis is the fact that a two testlet solution is in fact just two items, and this may affect the power of the test of fit to the model. However, these two ‘items’ have a considerable score range and the resulting indicator on the RUMM2030 program showed that the power of the test of fit was ‘excellent’.

## Conclusion

The DASH and QuickDASH have been shown to satisfy Rasch model expectations in a RA population, having accounted for local dependency of items through a testlet approach. Consequently, their raw (and standardized) scores can be deemed a sufficient statistic at the ordinal level for upper limb functioning. Rasch-transformed interval scaled estimates are available for the calculation of change score and other mathematical and parametric procedures. Data from other conditions will need to be re-analyzed to ensure that the breach of the local independence solution is adequately dealt with before other aspects are considered. Calculations of SEM and SDD for PROMs should always be based upon an unbiased estimate of reliability, having taken care of the inflation caused by a breach of the local independence assumption associated with all summative scales.

## Additional file

Additional file 1: **Table S1.** Local dependency amongst items of the DASH-30. **Table S2.** Local dependency amongst items of the QuickDASH. (DOCX 21 kb)
