# Supplementary material for: Revisiting the disabilities of the arm, shoulder and hand (DASH) and QuickDASH in rheumatoid arthritis
Source: BMC Musculoskelet Disord. 2019 Jan 25;20:41. doi: 10.1186/s12891-019-2414-6 (PMC6347833; doi:10.1186/s12891-019-2414-6)
Supplement: Supplementary file 1 — Table S1. Local dependency amongst items of the DASH-30. Table S2. Local dependency amongst items of the QuickDASH. (DOCX 21 kb) [file 12891_2019_2414_MOESM1_ESM.docx]

**Supplementary Table Legends**

**Supplementary Table 1: Local dependency amongst items of the DASH-30**

**Supplementary Table 2: Local dependency amongst items of the QuickDASH**

**Supplementary Table 1: Local dependency amongst items of the DASH-30**

| Item | I0001 | I0002 | I0003 | I0004 | I0005 | I0006 | I0007 | I0008 | I0009 | I0010 | I0011 | I0012 | I0013 | I0014 | I0015 | I0016 | I0017 | I0018 | I0019 | I0020 | I0021 | I0022 | I0023 | I0024 | I0025 | I0026 | I0027 | I0028 | I0029 | I0030 |
| --- | --- | --- | --- | --- | --- | --- | --- | --- | --- | --- | --- | --- | --- | --- | --- | --- | --- | --- | --- | --- | --- | --- | --- | --- | --- | --- | --- | --- | --- | --- |
| I0001 |  |  |  |  |  |  |  |  |  |  |  |  |  |  |  |  |  |  |  |  |  |  |  |  |  |  |  |  |  |  |
| I0002 | 0,022 |  |  |  |  |  |  |  |  |  |  |  |  |  |  |  |  |  |  |  |  |  |  |  |  |  |  |  |  |  |
| I0003 | 0,098 | 0,241 |  |  |  |  |  |  |  |  |  |  |  |  |  |  |  |  |  |  |  |  |  |  |  |  |  |  |  |  |
| I0004 | -0,024 | 0,164 | 0,217 |  |  |  |  |  |  |  |  |  |  |  |  |  |  |  |  |  |  |  |  |  |  |  |  |  |  |  |
| I0005 | 0,173 | 0,014 | 0,247 | 0,067 |  |  |  |  |  |  |  |  |  |  |  |  |  |  |  |  |  |  |  |  |  |  |  |  |  |  |
| I0006 | -0,009 | -0,025 | 0,148 | -0,061 | 0,149 |  |  |  |  |  |  |  |  |  |  |  |  |  |  |  |  |  |  |  |  |  |  |  |  |  |
| I0007 | -0,017 | -0,18 | 0,015 | 0,022 | 0,058 | 0,104 |  |  |  |  |  |  |  |  |  |  |  |  |  |  |  |  |  |  |  |  |  |  |  |  |
| I0008 | -0,008 | -0,093 | -0,108 | 0,004 | -0,014 | -0,11 | 0,281 |  |  |  |  |  |  |  |  |  |  |  |  |  |  |  |  |  |  |  |  |  |  |  |
| I0009 | -0,065 | -0,072 | -0,056 | 0,116 | -0,047 | -0,061 | 0,22 | 0,146 |  |  |  |  |  |  |  |  |  |  |  |  |  |  |  |  |  |  |  |  |  |  |
| I0010 | -0,075 | -0,01 | 0,026 | 0,044 | 0,153 | 0,132 | 0,11 | 0,051 | 0,184 |  |  |  |  |  |  |  |  |  |  |  |  |  |  |  |  |  |  |  |  |  |
| I0011 | 0,259 | -0,116 | 0,006 | -0,034 | 0,245 | 0,089 | 0,277 | 0,099 | 0,039 | 0,308 |  |  |  |  |  |  |  |  |  |  |  |  |  |  |  |  |  |  |  |  |
| I0012 | -0,021 | -0,103 | 0,119 | -0,044 | 0,013 | 0,366 | 0,139 | 0,009 | -0,075 | -0,072 | 0,127 |  |  |  |  |  |  |  |  |  |  |  |  |  |  |  |  |  |  |  |
| I0013 | -0,123 | -0,013 | 0,041 | 0,11 | -0,063 | 0,099 | 0,182 | 0,007 | 0,122 | 0,092 | 0,116 | 0,162 |  |  |  |  |  |  |  |  |  |  |  |  |  |  |  |  |  |  |
| I0014 | -0,092 | -0,074 | -0,016 | -0,049 | -0,105 | 0,105 | 0,055 | -0,104 | 0,051 | -0,044 | 0,014 | 0,111 | 0,187 |  |  |  |  |  |  |  |  |  |  |  |  |  |  |  |  |  |
| I0015 | -0,157 | 0,101 | 0,036 | 0,1 | -0,101 | 0,178 | -0,001 | -0,138 | 0,129 | -0,03 | -0,138 | 0,109 | 0,254 | 0,262 |  |  |  |  |  |  |  |  |  |  |  |  |  |  |  |  |
| I0016 | 0,06 | 0,16 | 0,168 | 0,193 | 0,118 | 0,002 | 0,03 | 0,018 | -0,027 | 0,112 | 0,014 | -0,058 | 0,128 | -0,048 | 0,14 |  |  |  |  |  |  |  |  |  |  |  |  |  |  |  |
| I0017 | -0,01 | 0,118 | 0,188 | 0,063 | 0,044 | 0,018 | -0,02 | -0,03 | -0,028 | 0,112 | 0,013 | 0,008 | 0,007 | -0,171 | -0,078 | 0,02 |  |  |  |  |  |  |  |  |  |  |  |  |  |  |
| I0018 | 0 | -0,152 | -0,048 | -0,081 | 0,062 | 0,01 | 0,145 | 0,136 | 0,036 | 0,056 | 0,072 | 0,028 | -0,009 | -0,075 | -0,089 | -0,148 | 0,012 |  |  |  |  |  |  |  |  |  |  |  |  |  |
| I0019 | -0,068 | -0,103 | -0,039 | -0,137 | 0,044 | 0,031 | 0,091 | 0,101 | -0,052 | -0,046 | -0,097 | 0,212 | -0,016 | -0,013 | -0,087 | -0,09 | -0,075 | 0,472 |  |  |  |  |  |  |  |  |  |  |  |  |
| I0020 | -0,135 | 0,088 | -0,019 | 0,03 | -0,023 | -0,059 | -0,046 | 0,227 | -0,012 | 0,011 | -0,053 | -0,054 | 0,066 | -0,001 | 0,084 | 0,047 | 0,029 | -0,107 | -0,063 |  |  |  |  |  |  |  |  |  |  |  |
| I0021 | -0,116 | -0,088 | -0,194 | -0,178 | -0,11 | -0,171 | -0,074 | -0,082 | -0,156 | -0,217 | -0,112 | 0,062 | -0,087 | 0,022 | -0,108 | -0,216 | -0,069 | -0,123 | 0,013 | 0,048 |  |  |  |  |  |  |  |  |  |  |
| I0022 | -0,167 | -0,109 | -0,232 | -0,008 | -0,277 | -0,221 | -0,163 | -0,115 | 0,017 | -0,121 | -0,215 | -0,233 | -0,154 | -0,073 | -0,029 | -0,043 | -0,191 | -0,122 | -0,084 | -0,016 | -0,008 |  |  |  |  |  |  |  |  |  |
| I0023 | -0,123 | -0,064 | -0,114 | -0,045 | -0,246 | -0,136 | -0,101 | -0,145 | 0,002 | -0,088 | -0,028 | -0,261 | -0,129 | -0,113 | -0,158 | -0,064 | -0,067 | -0,046 | -0,071 | -0,043 | -0,099 | 0,473 |  |  |  |  |  |  |  |  |
| I0024 | -0,058 | -0,04 | -0,131 | -0,038 | -0,14 | -0,234 | -0,318 | -0,218 | -0,154 | -0,162 | -0,25 | -0,345 | -0,246 | -0,242 | -0,1 | 0,002 | -0,071 | -0,149 | -0,161 | -0,11 | -0,025 | 0,281 | 0,225 |  |  |  |  |  |  |  |
| I0025 | -0,091 | 0,017 | -0,134 | -0,024 | -0,112 | -0,2 | -0,183 | -0,132 | -0,08 | -0,066 | -0,145 | -0,227 | -0,183 | -0,232 | -0,114 | -0,068 | -0,125 | -0,108 | -0,041 | -0,119 | -0,077 | 0,201 | 0,241 | 0,567 |  |  |  |  |  |  |
| I0026 | -0,069 | -0,086 | -0,157 | -0,115 | -0,202 | -0,16 | -0,153 | -0,06 | -0,147 | -0,094 | -0,134 | -0,141 | -0,106 | -0,056 | -0,122 | -0,131 | -0,016 | -0,183 | -0,134 | -0,072 | 0,086 | 0,127 | -0,043 | 0,066 | -0,032 |  |  |  |  |  |
| I0027 | -0,043 | 0,018 | -0,058 | -0,152 | -0,052 | -0,102 | -0,257 | -0,177 | -0,154 | -0,189 | -0,179 | -0,212 | -0,268 | -0,166 | -0,183 | -0,056 | -0,07 | -0,144 | -0,126 | -0,153 | -0,005 | 0,165 | 0,213 | 0,229 | 0,247 | 0,12 |  |  |  |  |
| I0028 | -0,061 | -0,037 | -0,106 | -0,14 | -0,165 | -0,163 | -0,308 | -0,189 | -0,186 | -0,244 | -0,228 | -0,192 | -0,242 | -0,086 | -0,069 | -0,121 | -0,117 | -0,151 | -0,137 | -0,134 | -0,039 | 0,175 | 0,117 | 0,439 | 0,364 | 0,087 | 0,333 |  |  |  |
| I0029 | -0,096 | -0,08 | -0,121 | -0,12 | -0,112 | -0,197 | -0,318 | -0,111 | -0,115 | -0,196 | -0,325 | -0,263 | -0,211 | -0,114 | -0,142 | -0,146 | -0,075 | -0,054 | -0,124 | -0,09 | 0,059 | 0,13 | 0,074 | 0,354 | 0,151 | 0,092 | 0,164 | 0,237 |  |  |
| I0030 | -0,047 | -0,068 | -0,225 | -0,12 | -0,182 | -0,201 | -0,171 | -0,108 | -0,118 | -0,192 | -0,202 | -0,101 | -0,241 | -0,04 | -0,077 | -0,138 | -0,094 | -0,095 | -0,132 | -0,108 | 0,075 | 0,037 | 0,055 | 0,044 | 0,021 | 0,132 | 0,078 | 0,134 | 0,179 |  |

**Supplementary Table 2: Local dependency amongst items of the QuickDASH**

|  | Item | I0001 | I0007 | I0010 | I0014 | I0016 | I0017 | I0022 | I0023 | I0024 | I0026 | I0029 |
| --- | --- | --- | --- | --- | --- | --- | --- | --- | --- | --- | --- | --- |
| I0001 | I0001 |  |  |  |  |  |  |  |  |  |  |  |
| I0007 | I0007 | 0,002 |  |  |  |  |  |  |  |  |  |  |
| I0010 | I0010 | -0,088 | 0,151 |  |  |  |  |  |  |  |  |  |
| I0014 | I0014 | -0,107 | 0,117 | -0,022 |  |  |  |  |  |  |  |  |
| I0016 | I0016 | 0,048 | 0,067 | 0,127 | -0,03 |  |  |  |  |  |  |  |
| I0017 | I0017 | -0,03 | 0,003 | 0,081 | -0,175 | 0,017 |  |  |  |  |  |  |
| I0022 | I0022 | -0,286 | -0,192 | -0,207 | -0,151 | -0,105 | -0,269 |  |  |  |  |  |
| I0023 | I0023 | -0,205 | -0,113 | -0,15 | -0,169 | -0,122 | -0,135 | 0,411 |  |  |  |  |
| I0024 | I0024 | -0,138 | -0,351 | -0,243 | -0,33 | -0,057 | -0,141 | 0,174 | 0,141 |  |  |  |
| I0026 | I0026 | -0,209 | -0,241 | -0,174 | -0,148 | -0,225 | -0,083 | -0,018 | -0,163 | -0,057 |  |  |
| I0029 | I0029 | -0,174 | -0,339 | -0,273 | -0,177 | -0,208 | -0,157 | 0,006 | -0,05 | 0,285 | -0,019 |  |
